# Cell-cycle arrest in mature adipocytes impairs BAT development but not WAT browning, and reduces adaptive thermogenesis in mice

**DOI:** 10.1038/s41598-017-07206-8

**Published:** 2017-07-27

**Authors:** Yuko Okamatsu-Ogura, Keigo Fukano, Ayumi Tsubota, Junko Nio-Kobayashi, Kyoko Nakamura, Masami Morimatsu, Hiroshi Sakaue, Masayuki Saito, Kazuhiro Kimura

**Affiliations:** 1 Department of Biomedical Sciences, Graduate School of Veterinary Medicine, Hokkaido University, Sapporo, 060-0818 Japan; 2 Laboratory of Histology and Cytology, Graduate School of Medicine, Hokkaido University, Sapporo, 060-8638 Japan; 3 Pharmaceutical Research and Technology Institute, Kindai University, Osaka, 577-8502 Japan; 4 Department of Disease Control, Graduate School of Veterinary Medicine, Hokkaido University, Sapporo, 060-0818 Japan; 5 Department of Nutrition and Metabolism, Institute of Health Biosciences, Tokushima University Graduate School, Tokushima, 770-8503 Japan

## Abstract

We previously reported brown adipocytes can proliferate even after differentiation. To test the involvement of mature adipocyte proliferation in cell number control in fat tissue, we generated transgenic (Tg) mice over-expressing cell-cycle inhibitory protein p27 specifically in adipocytes, using the aP2 promoter. While there was no apparent difference in white adipose tissue (WAT) between wild-type (WT) and Tg mice, the amount of brown adipose tissue (BAT) was much smaller in Tg mice. Although BAT showed a normal cellular morphology, Tg mice had lower content of uncoupling protein 1 (UCP1) as a whole, and attenuated cold exposure- or β3-adrenergic receptor (AR) agonist-induced thermogenesis, with a decrease in the number of mature brown adipocytes expressing proliferation markers. An agonist for the β3-AR failed to increase the number of proliferating brown adipocytes, UCP1 content in BAT, and oxygen consumption in Tg mice, although the induction and the function of beige adipocytes in inguinal WAT from Tg mice were similar to WT mice. These results show that brown adipocyte proliferation significantly contributes to BAT development and adaptive thermogenesis in mice, but not to induction of beige adipocytes.

## Introduction

There are three types of adipocytes, white, brown, and beige, and they have contrasting physiological roles in energy metabolism^1, 2^. White adipocytes store triglycerides and supply fatty acids to other tissues as energy substrates, whereas brown adipocytes consume fatty acids for thermogenesis. The thermogenic activity of brown adipocytes depends on the mitochondrial uncoupling protein 1 (UCP1), which dissipates the proton gradient as heat. Beige adipocytes are an inducible type of UCP1-expressing adipocyte, which appear in white adipose tissue (WAT) following chronic adrenergic stimulation such as cold exposure or treatment with a β3 adrenergic receptor (AR) agonist. Although the gene expression pattern is distinct from that of brown adipocytes^3^, beige adipocytes possess similar thermogenic ability, at least *in vitro* ^4, 5^.

The number of adipocytes drastically changes under certain physiological conditions^6, 7^. For example, expansion of WAT occurs in obese animals due to an increase in the size (hypertrophy) and the number (hyperplasia) of adipocytes^8–11^. Hyperplasia of brown adipose tissue (BAT), accompanied by an increase in brown adipocyte number, occurs in response to cold stimulation. In addition, the appearance of beige adipocytes may cause a change in the number of adipocytes^12^.

Fat tissue contains stem cells that proliferate and differentiate into several kinds of cells^13, 14^, and it is widely accepted that hyperplasia of both WAT and BAT is due to the proliferation and differentiation of progenitor cells or preadipocytes^15–20^. However, it has been repeatedly suggested that proliferation of mature adipocytes may also be involved in adipose tissue hyperplasia. During WAT hyperplasia, isotope-labeled thymidine was incorporated not only into the preadipocyte fraction, but also, into the mature adipocyte fraction^21–23^, although they are possibly attributed to the coexisting of adipose stem cells in this fraction. Primary cultured adipocytes can proliferate even after differentiation^24, 25^. With regard to BAT, we recently reported that the cell proliferation marker Ki67 was detected on UCP1-positive brown adipocytes, suggesting that brown adipocytes proliferate even after differentiation^26^. Moreover, as the number of these cells increased after cold exposure, it is likely that the proliferation of mature brown adipocytes, in addition to the proliferation of preadipocytes, may contribute to BAT hyperplasia after cold exposure^26^.

Cyclin-dependent kinase (CDK) inhibitor 1B (p27; also known as p27^Kip1^ or Kip1) is an endogenous CDK inhibitor that belongs to the CDK interacting protein/kinase inhibitory protein (Cip/Kip) family. p27 prevents the activation of cyclin E-CDK2 or cyclin D-CDK4 complexes by binding to the complex, resulting in the inhibition of cell-cycle progression during the G1 phase^27–29^. Over-expression of p27 induces the inhibition of cell division in several cell types^30–33^, and systemic p27 knockout mice have larger bodies and more WAT than wild-type (WT) mice^34, 35^. Thus, p27 plays a regulatory role in the cell proliferation process of various types of cells, including adipocytes.

In the present study, to extend the idea of mature adipocyte proliferation and to elucidate its possible contribution to the development of adipose tissues, we analyzed both brown and beige adipose tissue, and the induction of beige adipocytes, using adipocyte-specific p27-over-expressing mice.

## Results

### aP2-p27 Tg mice show normal growth and WAT, but less BAT

**Figure 1:**
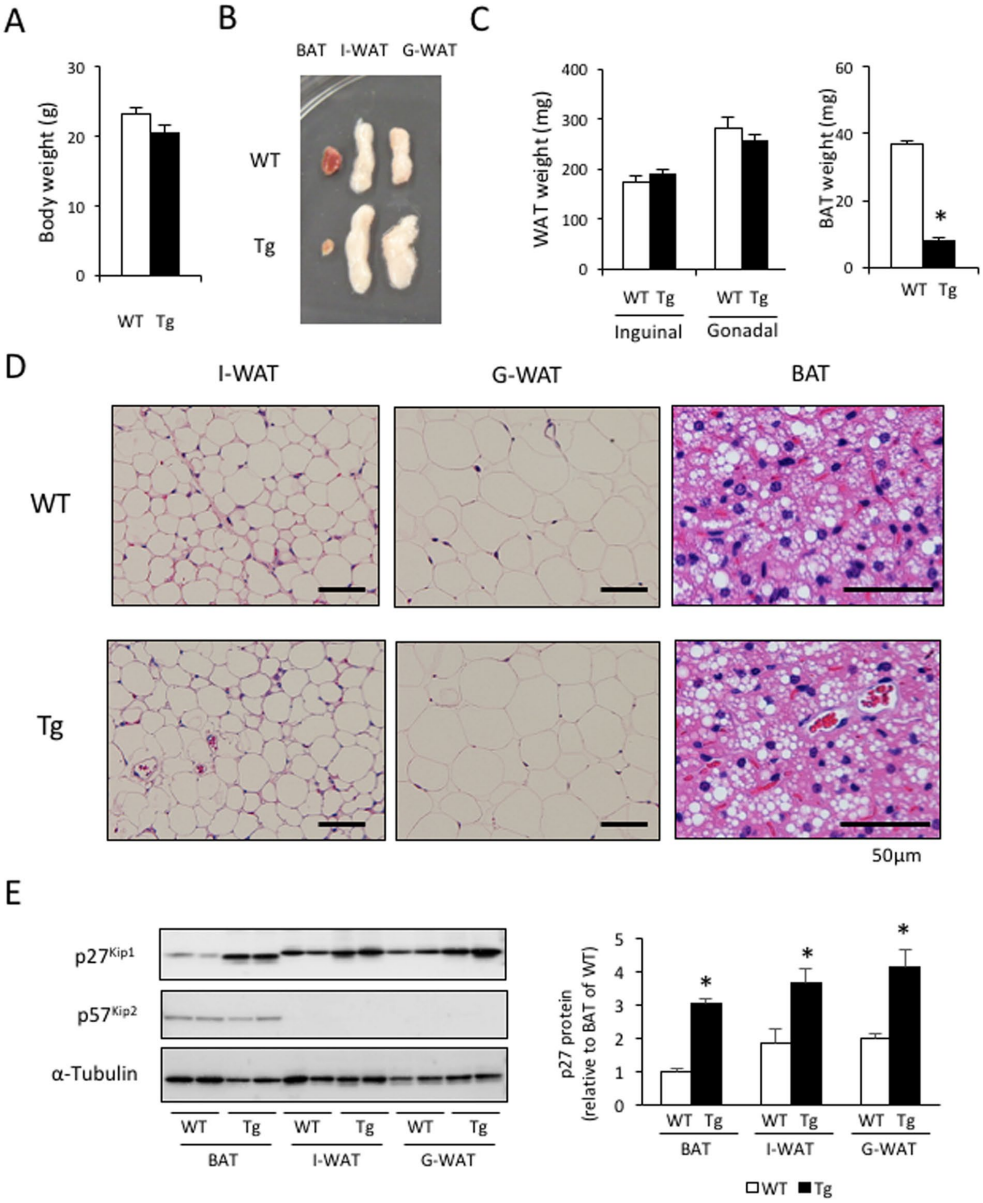
Transgenic expression of p27 driven by the aP2 promoter did not affect WAT development but suppressed BAT development. (**A**) There were no differences in body weight between 12-week-old WT and aP2-p27 Tg mice. (**B**–**D**) Tissue weights (**C**) and representative examples of the gross (**B**) and histological (**D**) appearance show that inguinal and peri-gonadal WAT (I-WAT and G-WAT) in Tg mice is highly similar to I-WAT and G-WAT in WT mice. Conversely, interscapular BAT in Tg mice was reduced in size and volume but had normal histological features, compared to BAT in WT mice (n = 5 per group, Student’s t-test, *p < 0.05). (**E**) Measurement of the expression of p27^Kip1^ and p57^Kip2^ proteins showed the induction of p27 protein in the fat tissue of Tg mice (n = 5 per group, Student’s t-test, *p < 0.05).

To investigate whether the inhibition of cell-cycle progression in mature adipocytes affects the development of adipose tissue, transgenic (Tg) mice expressing p27 under the control of the adipocyte-specific aP2 promoter were generated. Tg mice were born in accordance with the Mendelian ratio, and grew normally to adulthood. At 12 weeks, the body length and weight of the Tg mice were 8.6 ± 0.0 cm and 20.6 ± 0.7 g, respectively, and there were no significant differences compared to WT mice (8.7 ± 0.1 cm and 23.2 ± 0.6 g) (Fig. 1A). The gross and histological appearance, or the weight, of inguinal WAT (I-WAT) and perigonadal WAT (G-WAT) in Tg mice were not different to those of WT mice (Fig. 1B and C). However, in contrast to WAT, the amount of interscapular BAT in Tg mice was markedly reduced compared to WT mice (Fig. 1B and C). Despite this, the cellular morphology and lipid content of the BAT was almost identical in WT and Tg mice (Fig. 1D). Western blot analysis showed that p27 was expressed at similar levels in I-WAT and G-WAT, but at lower levels in interscapular BAT, which were approximately 52% of that in the I-WAT of WT mice (Fig. 1E). p27 expression in BAT, I-WAT, and G-WAT in Tg mice was increased 3.1-, 2.0-, and 2.1- fold, respectively, compared to that in WT mice. In BAT, there was no difference in the expression of p57^Kip2^, another CDK-inhibitor which shares the same degradation pathway via the SCF/Skp2^36^, although p57^Kip2^ protein was not detected in I-WAT and G-WAT.

These results indicate that over-expression of p27 in mature adipocytes has no effect on WAT development, but suppresses BAT development.

### aP2-p27 Tg mice show lower content of UCP1 in BAT and attenuated cold exposure- or β3-AR agonist-induced thermogenesis

**Figure 2:**
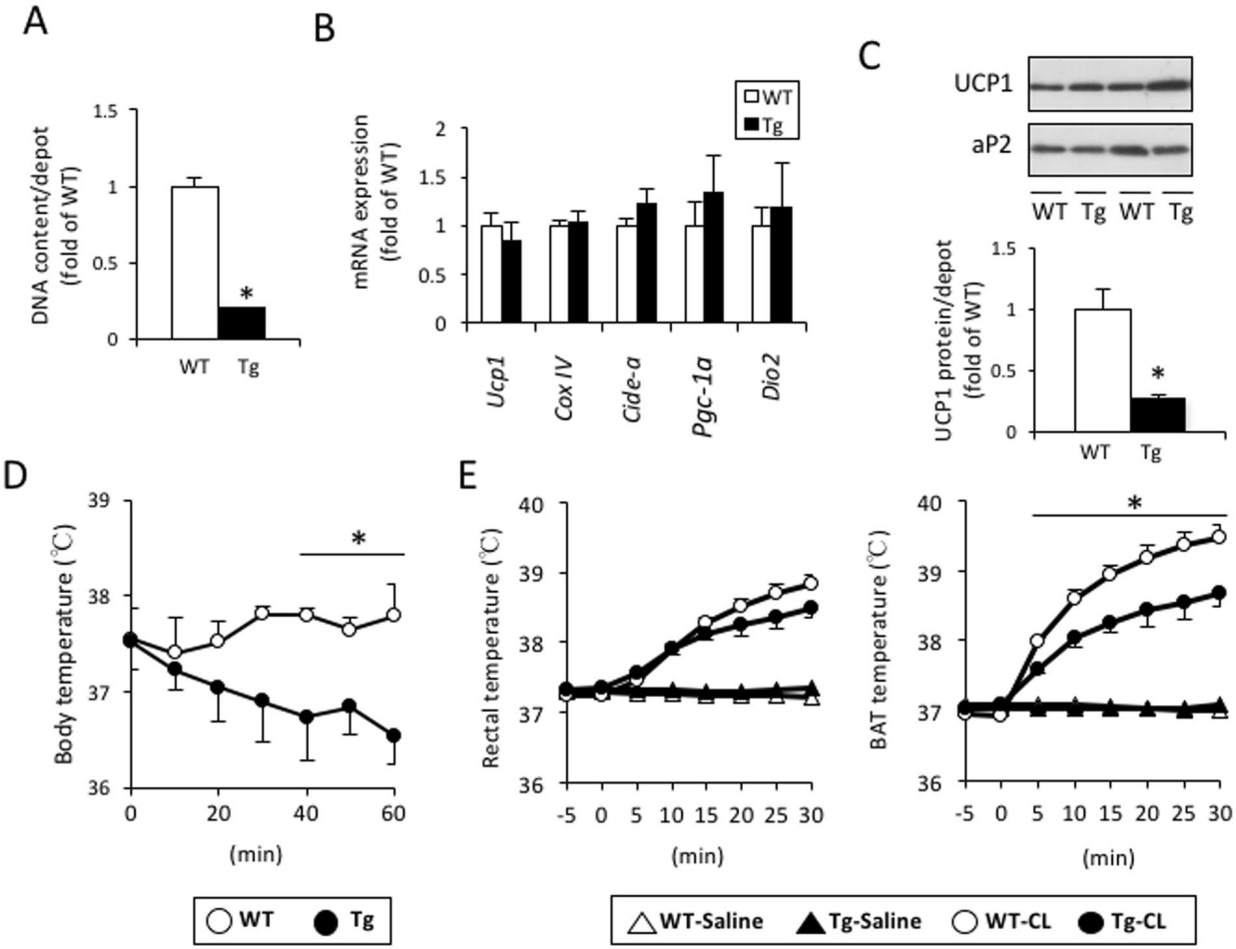
aP2-p27 transgenic mice had a lower amount of UCP1 and an attenuated thermogenic response to cold exposure or β3-AR agonism. (**A**) The total amounts of DNA per BAT in Tg mice were reduced compared to those in WT mice (n = 5 per group, Student’s t-test, *p < 0.05). (**B** and **C**) Quantification of mRNA expression in BAT from WT and Tg mice (**B**) revealed a comparable expression of genes related to thermogenic function. The total amounts of UCP1 per the BAT as a whole (**C**) in Tg mice were reduced, compared to those observed in WT mice (n = 5 per group, Student’s t-test, *p < 0.05). (**D**) Core body temperatures in Tg mice were significantly decreased after exposure to an ambient temperature of 4 °C (n = 5 per group, one-way ANOVA followed by the Tukey–Kramer *post-hoc* test, *p < 0.05) while these were maintained in WT mice. (**E**) Rectal and interscapular BAT temperatures were increased by i.p. injection of CL316,243 (0.1 mg/kg) in Tg mice under urethane anesthesia, but the CL316,243-induced increase in BAT temperature was attenuated compared to that seen in WT mice (n = 5 per group, one-way ANOVA followed by the Tukey–Kramer *post-hoc* test, *p < 0.05).

As neither cell size nor the lipid content in brown adipocytes differed between WT and Tg mice (Fig. 1D), the smaller amount of BAT in Tg mice could be due to a reduction in the number of adipocytes. In this regard, DNA content per the whole tissue in the BAT of Tg mice was significantly lower than in WT mice (Fig. 2A). The mRNA expression levels of several genes related to BAT thermogenic function (that is, *Ucp1*, *CoxIV Cide-a*, *Pgc-1a*, and *Dio2*) in Tg mice were comparable to those in WT mice (Fig. 2B). Consistent with the mRNA expression data, thermogenic UCP1 levels per mg protein in Tg mice were similar to those in WT mice (Fig. 2C). However, the total amount of UCP1 per the whole tissue was only 27.5% of that measured in WT mice (Fig. 2C), presumably because of the reduced number of brown adipocytes.

To evaluate the thermogenic function of BAT in Tg mice, responses to cold exposure and the β3-AR agonist CL316,243 (CL) were examined. The baseline body temperatures of WT and Tg mice were 37.6 °C ± 0.3 °C and 37.5 °C ± 0.3 °C, respectively. When exposed to a cold ambient temperature of 4 °C, the rectal temperatures of the Tg mice gradually fell, resulting in a 1.0 °C ± 0.2 °C drop in 60 min, whereas body temperature in WT mice was maintained at a constant level (Fig. 2D). CL injection produced a rapid rise in both BAT and rectal temperatures, with BAT temperature being higher than rectal temperature at 10 min after the CL injection and thereafter both in WT and Tg mice, indicating the rise in rectal temperature was driven by the rise of BAT temperature (Fig. 2E). However, the response to CL injection was weaker in Tg mice.

These results indicate that the thermogenic capability of BAT in Tg mice was reduced as a whole compared to that of WT mice, presumably due to the impairment of BAT development.

### aP2-p27 Tg mice show reduced brown adipocyte proliferation

**Figure 3:**
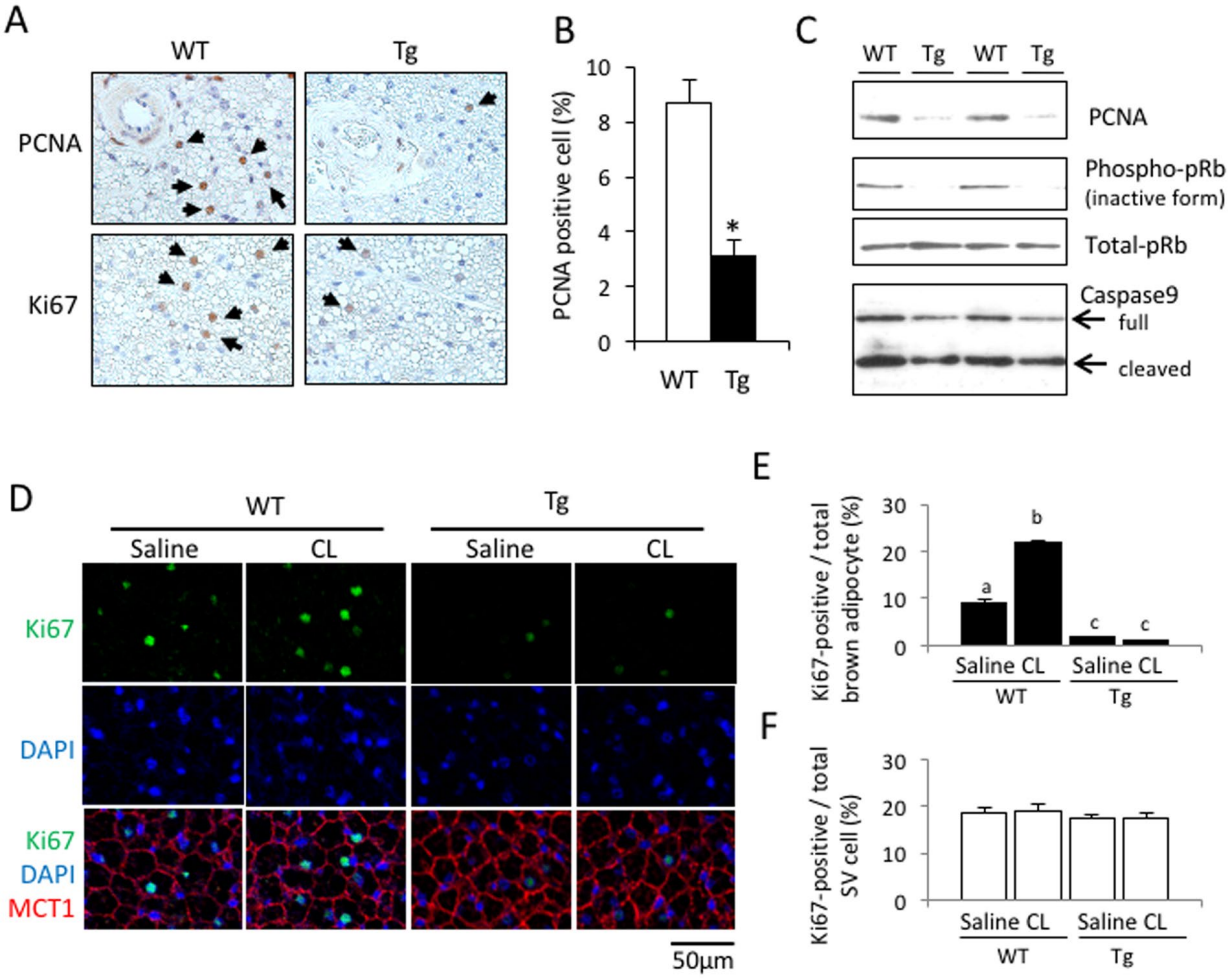
Transgenic expression of p27 driven by the aP2 promoter suppressed cell proliferation in BAT. (**A**–**C**) Representative images of BAT sections from Tg mice show a lower expression of the cell proliferation markers, PCNA and Ki67, compared to WT mice (**A**). Quantification of PCNA-positive cells (**B**) and western blot analyses (**C**) in the BAT confirmed decreased expression of proliferative markers but there were no essential differences with respect to the apoptosis-related protein (n = 5 per group, Student’s t-test, *p < 0.05). (**D**–**F**) Mice were injected with the β3-adrenergic agonist CL316,243 (CL; 0.1 mg/kg, s.c.) and BAT was sampled 24 h later. Representative images of BAT sections stained with the Ki67 antibody, along with the MCT1 antibody and DAPI, (**D**) identified mature brown adipocytes as having round nuclei separated from the MCT1-positive cell membrane, and stromal-vascular (SV) cells as having flattened nuclei sandwiched between the MCT1-positive cell membranes^26^. Graphs representing the quantified data show that the number of Ki67-positive brown adipocytes (**E**), but not the number of SV cells (**F**), was much lower in Tg mice, and an increase after CL treatment was observed only in WT mice (n = 4 per group, one-way ANOVA followed by the Tukey–Kramer *post-hoc* test, different letters indicate significant differences between groups, p < 0.05).

As p27 is known to arrest cell-cycle progression, it is conceivable that the over-expression of p27 in brown adipocytes mediated by the aP2 promoter could directly affect their proliferation, resulting in a smaller amount of BAT. Immunohistochemical analysis showed that the number of proliferating cells stained with proliferating cell nuclear antigen (PCNA) and Ki67 antibodies in the BAT of Tg mice was only one-third of the number observed in WT mice (Fig. 3A and B). Western blot analysis also confirmed a marked reduction in PCNA expression in the BAT of Tg mice (Fig. 3C). Moreover, phosphorylated retinoblastoma protein, an inactive form of the tumor-suppressing retinoblastoma protein, was only detected in WT mice, despite non-phosphorylated retinoblastoma protein being present in similar concentrations in Tg and WT mice (Fig. 3C). There was no significant difference in caspase-9 expression between the BAT of Tg and WT mice (Fig. 2C). Note that no positive signal was obtained by TUNEL staining, and expressions of the cleaved forms of caspase-3 and -6 were not detected in BAT of both mice. These results collectively suggest that impairment of BAT development by p27 over-expression is due to a decrease in cell proliferation but not an increase in apoptosis.

Previously, we reported that cold exposure or CL administration increases the number of Ki67-expressing proliferating mature brown adipocytes in mice^26^. Since Tg mice were cold intolerant (Fig. 2D), we injected both mice with CL and examined the effect on Ki67 expression in BAT 24 h later (Fig. 3D). Monocarboxylate transporter 1 (MCT1), a membrane protein expressed abundantly in brown adipocytes^37^, was used to distinguish brown adipocytes from stromal vascular (SV) cells in BAT sections, as previously reported^26^. In saline-injected control mice, Ki67-positive brown adipocytes were observed in the BAT of WT and Tg mice, but the number of cells was significantly lower in Tg mice (1.5% ± 0.1% of the total number of brown adipocytes) compared to WT mice (8.9% ± 0.9%) (Fig. 3E). CL injection increased the number of Ki67-positive brown adipocytes 2.4-fold in WT mice, but no effect was observed in Tg mice. In contrast, the number of Ki67-positive SV cells was similar in both Tg and WT mice following saline injection, and this was not altered by CL injection (Fig. 3F). These results also confirm that the proliferation of mature brown adipocytes is arrested in Tg mice.

The localization of p27 in BAT of WT and Tg mice was also examined by the immunofluorescent staining for p27 and MCT1. As shown in Supplementary Figure 1A, p27 protein was detected in cytoplasm and nucleus of brown adipocyte, with higher intensity in nucleus both in WT and Tg mice, and the expression level was much higher in Tg mice than in WT mice. In BAT of Tg mice, p27-positive nuclei were observed in mature brown adipocytes (Supplementary Fig. S1B, arrowheads), but not in SV cells (Supplementary Fig. S1B, arrows). These results confirmed that transgenic expression of p27 is restricted in mature brown adipocytes and not expressed in SV cells in BAT of Tg mice.

### Over-expression of p27 fails to affect the differentiation of brown adipocytes *in vitro*

**Figure 4:**
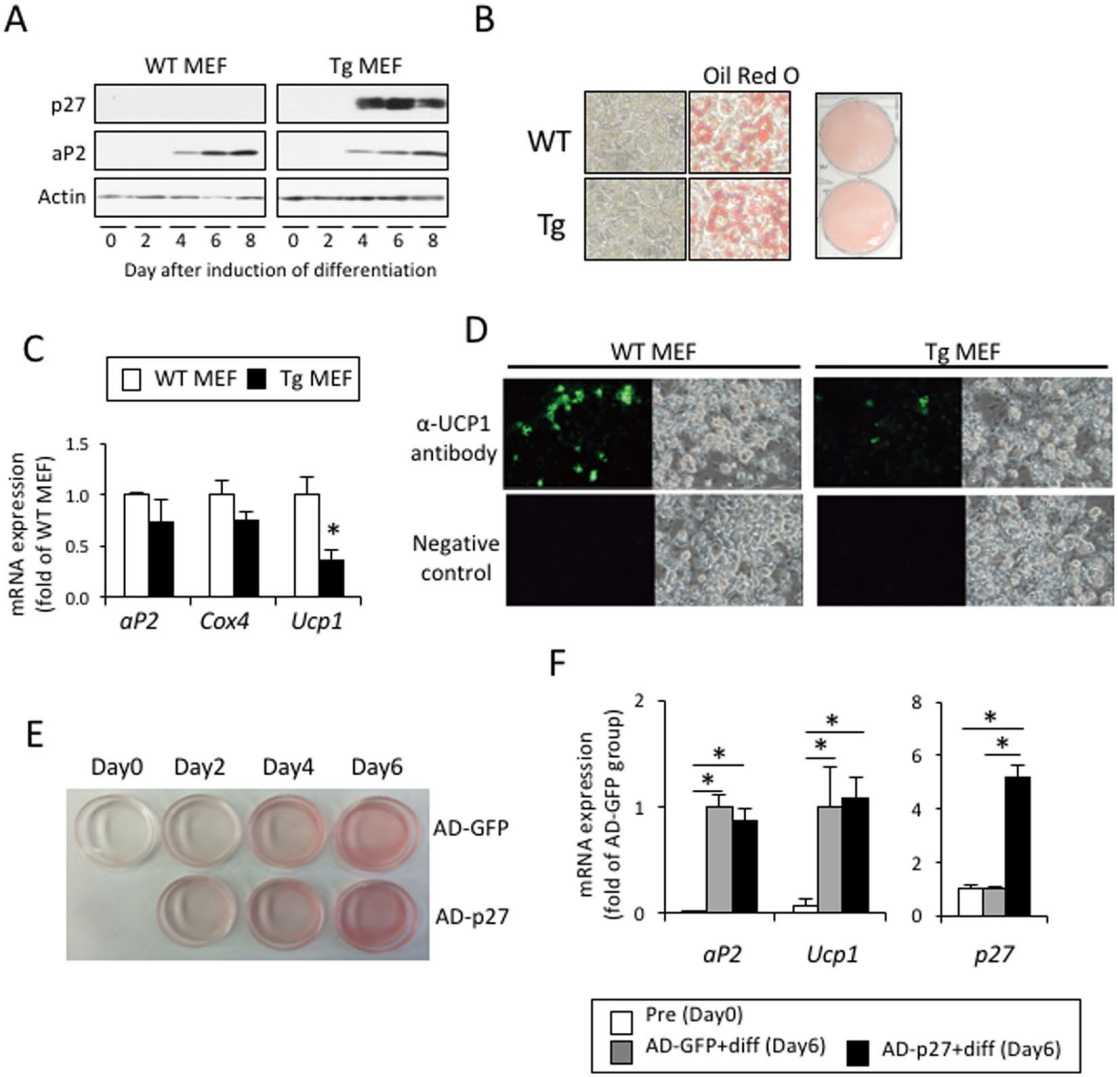
Expression of p27 has no effect on adipocyte differentiation. (**A**–**D**) Mouse embryonic fibroblasts (MEFs) prepared from WT or aP2-p27 Tg mice embryos at day 16.5 of gestation were cultured and differentiated into adipocytes by treatment with 0.5 mM IBMX and1 µM Dex for the first two days, and subsequently with 10 µg/ml insulin, 50 nM T3, and 10 µM Tro for six days. Assessment of the expression of aP2 during differentiation (**A**,**C**) and cellular lipid staining with Oil Red O at Day 8 (**B**) showed that there were no differences in adipocyte differentiation between the MEFs of WT and Tg mice. Conversely, the expression of UCP1 mRNA (**C**) and the number of UCP1-expressing cells (**D**) were significantly decreased in MEFs from Tg mice, compared to WT mice, and accompanied by increased p27 expression (**A**) (n = 4 per group, Student’s t-test, *p < 0.05). (**E** and **F**) HB2 cells were infected with adenovirus expressing GFP (AD-GFP) or p27 (AD-p27), and were then differentiated by treatment with 10 µg/ml insulin and 50 nM T3 for six days. Cellular lipid staining (**E**) and quantification of the expression of aP2 and UCP1 (F, n = 4 per group, one-way ANOVA followed by the Tukey–Kramer *post-hoc* test, *p < 0.05) showed that the forced expression of p27 had no effect on adipocyte differentiation in HB2 cells.

The effects of over-expression of p27 were also examined in cultured mouse embryonic fibroblasts (MEFs) prepared from both WT and Tg mice. p27 was not detected in fibroblasts, but was instead induced four days after adipogenic differentiation, accompanied by increased aP2 expression (Fig. 4A). This p27 induction was not observed in the cultured MEFs from WT mice. After eight days of culture, MEFs from Tg and WT mice differentiated to adipocytes, which contained comparable lipid droplets (Fig. 4B) and expressed similar levels of *aP2* and *Cox4*, an index of the mitochondrial number (Fig. 4C). However, UCP1 mRNA expression in the MEFs of Tg mice was significantly lower compared to that in the MEFs of WT mice, probably due to a reduction in the number of UCP1-expressing cells (Fig. 4D). To test the effects of p27 expression on adipocyte differentiation more directly, we analyzed the pre-brown adipocyte HB2 cell line infected with an adenovirus carrying the GFP (AD-GFP) or p27 (AD-p27) gene. HB2 cells infected with AD-p27 differentiated into adipocytes and expressed aP2 and UCP1 in a similar fashion to those cells infected with AD-GFP (Fig. 4E and F), despite a large difference in the p27 expression level (Fig. 4F). Thus, p27 expression may not affect the differentiation process of adipocytes.

### aP2-p27 Tg mice show normal beige adipocyte generation in WAT, but fail to increase energy expenditure after chronic β3-AR agonist treatment

**Figure 5:**
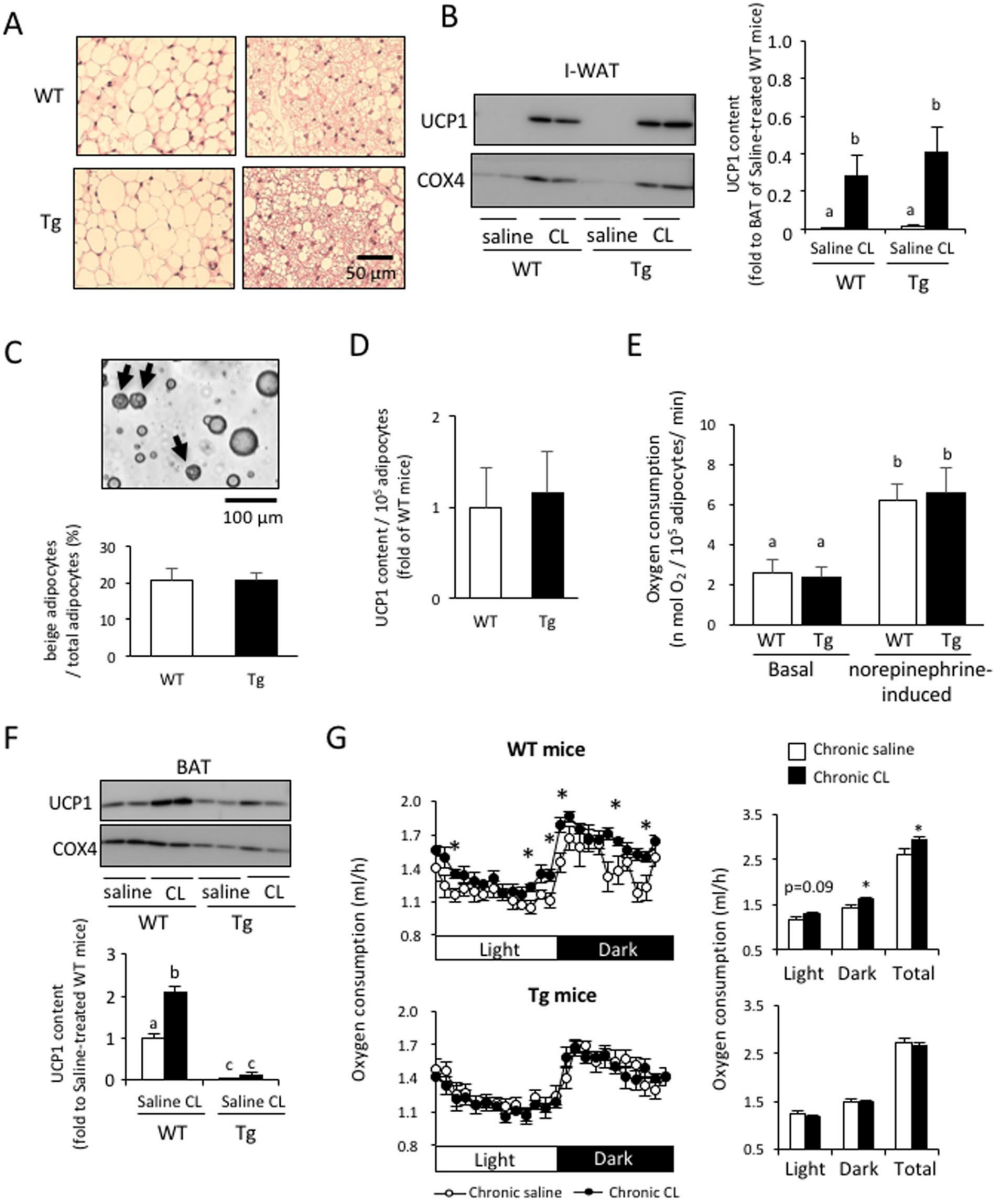
Transgenic expression of p27 driven by the aP2 promoter did not affect the generation and function of beige adipocytes. Mice acclimated to a room temperature of 28 °C were injected with saline or CL316,243 (CL; 1 mg/kg/day, s.c.) for 14 days. (**A** and **B**) Representative histological images (**A**) and measurement of the UCP1 content per inguinal-WAT (I-WAT) (**B**) show that there were no differences between WT and Tg mice with regard to the induction of beige adipocytes by CL. (**C**–**E**) Analysis of adipocyte fractions isolated from the I-WAT of WT and Tg mice after CL treatment showed the presence of a similar number of multilocular beige adipocytes (**C**), similar levels of UCP1 (**D**), and consumption of a similar amount of oxygen in either the basal or norepinephrine-induced state (**E**). (**F**) Chronic CL treatment failed to increase levels of UCP1 per BAT in Tg mice. (n = 6 per group, one-way ANOVA followed by the Tukey–Kramer *post-hoc* test, different letters indicate significant differences between groups, p < 0.05). (**G**) Enhancement of oxygen consumption after chronic CL treatment was observed in WT mice but not in Tg mice (n = 6 per group, Student’s t-test, *p < 0.05).

To examine the effects of the inhibition of adipocyte proliferation on beige adipocyte generation, WT and Tg mice were administered CL for two weeks. To exclude the effects of environmental temperature, experiments were conducted at 28 °C. In both WT and Tg mice, CL treatment induced multilocular beige adipocytes (Fig. 5A) and increased UCP1 content per the whole tissue (Fig. 5B) in I-WAT, but there was no difference in UCP1 levels between WT and Tg mice (Fig. 5B). When adipocytes were isolated from the I-WAT of mice using a collagenase digestion method, there were no differences in the ratio of multilocular beige adipocytes to the total number of adipocytes, the UCP1 content per 10^5^ adipocytes, or basal and norepinephrine-induced oxygen consumption, between WT and Tg mice (Fig. 5C–E). Thus, the generation and the function of beige adipocytes were not affected by adipocyte-specific p27 expression, indicating that beige adipocyte generation does not require mature adipocyte proliferation.

Although chronic CL treatment brought about a similar increase in UCP1 content in I-WAT in WT and Tg mice, it increased BAT UCP1 content per the whole tissue in WT mice only (Fig. 5F), reflecting the greatly attenuated CL-induced proliferation of brown adipocytes observed in Tg mice (Fig. 3D). It is worth noting that, compared to the BAT of WT mice, and even after CL treatment, UCP1 content per the whole tissue in I-WAT was as low as 28.6% and 40.7% in WT and Tg mice, respectively (Fig. 5B).

To examine how the increase in UCP1 in BAT and I-WAT affects whole-body energy expenditure, oxygen consumption was measured (Fig. 5G). In WT mice, chronic CL treatment resulted either in a tendency towards an increase or a significant increase in basal oxygen consumption in the light and dark phases. Total oxygen consumption was 12.8% higher in the CL-treated group than in the saline-treated group. In contrast, oxygen consumption was not altered by CL treatment in Tg mice. These results indicate that the increase in UCP1 in BAT, rather than UCP1 in I-WAT, significantly contributes to basal oxygen consumption.

## Discussion

Adipocyte numbers in fat tissue can drastically change, depending on the physiological context^3, 4^. During WAT hyperplasia, mature adipocytes, in addition to preadipocytes, arguably contribute to an increase in cell number, although mature adipocytes are post-mitotic terminally-differentiated cells^21–23^. Here using Tg mice with over-expression of p27 in mature adipocytes under the control of the aP2 promoter, we found that the WAT of Tg mice was similar to that of WT mice, both in weight and morphology. This suggests that mature adipocyte proliferation, if present, is not necessary for the development of WAT. In contrast, the BAT of Tg mice was considerably different to that of WT mice, there being a markedly smaller amount of tissue with reduced expression of proliferating signals. As p27 appeared to have a minor effect on the differentiation of brown adipocytes, at least *in vitro*, the reduced amount of the BAT could be attributed to impaired proliferation of adipocytes by the inhibitory action of p27. These results thus confirm our previous findings that mature brown adipocytes can proliferate even after differentiation. The difference in the proliferative ability between brown and white adipocytes cannot be explained at present, but it could be due to their different origins: the former arise from myogenic factor 5 (Myf5)-positive progenitors, while the latter stem from Myf5-negative cells^38^. In support of this, the generation of beige adipocytes, which have been reported to arise from Myf5-negative cells along with white adipocytes^38^, was not affected by p27 expression.

Previously, we reported that mature brown adipocytes proliferate after cold exposure *via* a β3-AR-mediated pathway, resulting in BAT hyperplasia^26^. Indeed, proliferation of brown adipocytes was induced by the injection of the β3-AR agonist CL in WT mice, a response that was greatly attenuated in Tg mice. Long-term CL treatment increased UCP1 content in the BAT of WT mice, but not in that of Tg mice, indicating that this increase is partly due to an elevation in the number of brown adipocytes, although UCP1 gene expression in each brown adipocyte was also enhanced. Interestingly, chronic CL treatment increased oxygen consumption in WT mice, but not in Tg mice. As the induction and the function of beige adipocytes were similar in WT and Tg mice, we can conclude that BAT hyperplasia significantly contributes to the adaptive thermogenesis seen after chronic CL treatment.

Our study also revealed the minor contribution of beige adipocytes in adaptive thermogenesis. We and others have previously reported that beige adipocytes have a similar thermogenic ability to classical brown adipocytes *in vitro* ^4, 5^. The minor role of beige adipocytes in whole-body energy expenditure may be due to the low UCP1 content in WAT, even when beige adipocytes are maximally induced, compared to the UCP1 content in classical BAT in mice^39^. On the other hand, the emergence of beige adipocytes in WAT has been reported to be associated with a lean phenotype in several lines of transgenic mice^40^. One possible explanation is that beige adipocytes control adiposity through some mechanism other than by increasing energy expenditure; for example, through the secretion of adipokines^41^. However, it is more plausible that the obesity-resistant phenotypes of these transgenic mice are due to the enhanced thermogenic ability of BAT, as it is often the case that UCP1 content is increased not only in WAT, but also in BAT, in such mouse lines. However, total UCP1 content per the whole WAT or BAT, and their contribution to UCP1 amount per whole body have not been estimated in these mice. Thus, it is difficult to estimate the role of beige adipocytes in isolation, with no regard for the possible contribution of BAT, in whole-body energy expenditure or body fat control. In this study, we found that aP2-p27 Tg mice have considerably smaller amounts of BAT, but show normal ability in terms of beige adipocyte induction. When studied at 28 °C, UCP1 content in the BAT of Tg mice was as little as 13% of that seen in WT mice, even after chronic CL treatment, whereas CL-induced UCP1 content in the I-WAT of Tg mice was similar to that measured in WT mice. This mouse model would be useful to examine the function of beige adipocytes *in vivo*.

In summary, we demonstrated that brown adipocytes have a unique feature of retaining proliferative ability after differentiation, which is required for the development of BAT, but not for the development or browning of WAT. Furthermore, using a new unique model of mice that have extremely small amounts of BAT but normal WAT browning ability, we demonstrated that classical brown adipocytes, rather than beige adipocytes, have an important role in adaptive thermogenesis after chronic CL treatment. Our findings may provide new insight into the mechanism and function of BAT recruitment.

## Materials and Methods

### Materials

Dulbecco’s modified Eagle’s medium (DMEM)-high glucose and collagenase were purchased from Wako Pure Chemicals (Osaka, Japan). Fatty acid-free bovine serum albumin (BSA), isobutylmethylxanthine (IBMX), dexamethasone (Dex), insulin, 3,3′,5-triiodothyronine (T3), and troglitazone (Tro) were purchased from Sigma-Aldrich (St. Louis, MO, USA). Fetal calf serum (FCS) was obtained from Trace Scientific Ltd. (Melbourne, Australia). Rabbit anti-serum against UCP1 was a gift from Dr. Teruo Kawada (Kyoto University, Japan). Antibodies against aP2, caspase-9, and the phosphorylated form of Rb were purchased from Cell Signaling Technology (Beverly, MA, USA); those against β-actin, p57, and a-Tubulin were from Sigma-Aldrich; those against p27 and Rb were from BD Biosciences (San Jose, CA, USA); that against PCNA was from Santa Cruz Biotechnology (Santa Cruz, CA, USA); and those against Ki67 and MCT1 were from Abcam (Cambridge, MA, USA). Anti-rabbit and anti-mouse secondary antibodies conjugated with horseradish peroxidase were obtained from Cell Signaling Technology. Anti-rabbit or anti-chicken secondary antibodies conjugated with Alexa Fluor 488 and Alexa Fluor 594 were obtained from Thermo Fisher Scientific (Gaithersburg, MD, USA).

### Animals

The experimental procedures and care of animals were approved by the Animal Care and Use Committee of Hokkaido University (Hokkaido, Japan). All experiments using mice were performed in accordance with the guidelines of Hokkaido University Manual for Implementing Animal Experimentation, in the animal facility approved by the Association for Assessment and Accreditation of Laboratory Animal Care (AAALAC) International. To generate aP2-p27 transgenic mice, mouse p27 cDNA (GenBank accession NM_009875.4) was cloned into downstream of the 5.4-kb mouse aP2 promoter/enhancer and intron of rabbit beta-globlin, and upstream of a human GH poly (A) sequence^42^. The transgene construct was microinjected into fertilized mouse C57BL/6 J oocytes. The transgenic founder lines were maintained as heterozygous animals. Mice were housed in plastic cages within an air-conditioned room at 23 °C with a 12:12 h light:dark cycle and given free access to laboratory chow (Oriental Yeast, Tokyo, Japan) and tap water. Mice were euthanized at 12 weeks old using carbon dioxide, and interscapular BAT and I- and G-WAT were promptly removed and weighed. Tissue specimens were fixed in 10% buffered formalin or 4% paraformaldehyde for histological examination, or transferred into liquid nitrogen for western blot analysis and the measurement of DNA content. In experiment of acute effect of β3-AR agonist CL316,243 (CL; American Cyanamid, Pearl River, NY, USA) on the expression of proliferation marker, mice were injected subcutaneously with saline or CL (0.1 mg/kg), and euthanized using carbon dioxide 24 h later. BAT was sampled and fixed in Bouin’s fluid.

### Measurement of body and tissue temperature

Body temperature was measured in conscious, unrestrained mice using the VitalView Data Acquisition System (Mini-Mitter Co., Sunriver, OR, USA). Briefly, a transmitter (PDT-4000) (Mini-Mitter Co.) was implanted into the peritoneal cavity of a mouse under anesthesia by intraperitoneal injection with 75 mg/kg ketamine and 1 mg/kg medetomidine. Mice were allowed to recover for a minimum of seven days after implantation. Signals from the transmitter were monitored by a receiver (ER-4000) (Mini-Mitter Co.). After a 1-h measurement of baseline body temperature, mouse cages were transferred to a room with an ambient temperature of 4 °C, and body temperature was monitored for 1 h.

Tissue temperature was measured as previously reported, with a slight modification^43^. Briefly, mice were anesthetized with urethane (1 g/kg, intraperitoneal, i.p.), and a small incision was made above the scapula. The interscapular brown fat pads were partially separated from the muscle below, with the vasculature and nerve supplies to the pads left intact. Mice were then placed on a hot plate and the plate was heated gently to maintain rectal temperature stabilized at approximately 37 °C. A plastic-coated thermistor with a diameter of 1 mm was placed under the fat pads. Another thermistor was also inserted into the rectum. β3-AR agonist CL (0.1 mg/kg) or saline was injected intraperitoneally, and the resulting temperature changes were monitored for 30 min.

### Chronic treatment with CL and oxygen consumption measurement in mice

Mice were acclimated to room temperature at 28 °C for one week, and then injected with CL (0.1 mg/kg) or saline subcutaneously (s.c.) once a day at 19:00 for 14 days. The day after the last injection, mice were transferred to a transparent chamber (50W × 150D × 150H mm), and oxygen consumption was measured after the adaptation period for at least 24 h. This was carried out using an O_2_ metabolism measuring system (model MM102R; Muromachi Kikai, Tokyo, Japan) for 1 min at 3-minute intervals for 1 h, and expressed as ml O_2_ per mouse. Mice had free access to food and water during the measurement.

### Isolation and oxygen consumption measurement of adipocytes

I-WAT was minced into small pieces and digested in Krebs–Ringer HEPES buffer (KRHB) containing fatty acid-free BSA (10 mg/ml), 2.5 mM glucose, and collagenase (1 mg/ml) for 1 h at 37 °C, with shaking at 90 cycles/min. The cell suspension was passed through a 200-µm nylon filter, and the filtrate was centrifuged at 50 × g for 2 min. The floating cells were washed three times with KRHB to eliminate collagenase, brought to a suitable dilution in KRHB containing 4% BSA and 2.7 mM glucose, and kept at room temperature for 1 h before use. The oxygen consumption of the isolated adipocytes was measured polarographically using a Clark-style oxygen electrode in a water-jacketed Perspex chamber at 37 °C. The isolated adipocytes were incubated in the chamber in 1 ml KRHB containing 4% BSA and 2.7 mM glucose. The oxygen concentration in the chamber was monitored continuously for 5 min before and 10 min after the addition of 1 µM norepinephrine. Oxygen consumption rates were calculated using computer software (782 System; Strathkelvin Instruments, Glasgow, Scotland).

### Preparation of MEFs and cell culture

MEFs were prepared from embryonic day 16.5 embryos. Briefly, embryos were minced in trypsin–EDTA (0.25%), and incubated for 45 min at 37 °C with pipetting every 15 min to disaggregate the tissue. After the addition of the culture medium (DMEM-high glucose containing 10% FCS), the cell suspension was passed through a 200 µm nylon filter and the filtrate was centrifuged at 200 × g for 5 min at room temperature. The pellet was suspended in hemolytic buffer (150 mM NH_4_Cl, 10 mM KHCO_3_, 0.1 mM Na_2_EDTA, pH 7.4), and passed through a 25-µm nylon filter. The filtrate was then centrifuged at 200 × g for 5 min at room temperature and the MEF cells were obtained as the pellet. MEF cells were maintained in the culture medium and were differentiated at 100% confluency by treatment with 0.5 mM IBMX and 1 µM Dex for two days; and 10 µg/ml insulin, 50 nM T3, and 10 µM Tro for six days, with the medium being changed every two days.

HB2 cells^44^ were infected with an adenovirus expressing GFP (AD-GFP) or p27 (AD-p27) at a multiplicity of infection (MOI) of 100 in FCS-free DMEM-high glucose medium for 12 h. After infection, cells were kept in a culture medium containing 10 mg/ml insulin and 50 nM T3 for the subsequent six days, with the medium being changed every two days.

### Immunohistochemical analysis

For the immunostaining of PCNA and Ki67 in BAT, deparaffinized sections of 5 µm thickness were incubated in 0.3% hydrogen peroxide in methanol. For the detection of Ki67, microwave-enhanced antigen retrieval was performed by boiling the slides in 10 mM citrate buffer (pH 6.0) containing 0.05% Tween 20. After washing with PBS, the slides were incubated for 1 h with 10% normal goat serum before incubation with the primary antibody (anti-PCNA, 1:300; anti-Ki67, 1:300) overnight at 4 °C. Slides were rinsed in PBS and incubated with biotin-labeled secondary antibody (Nichirei, Tokyo, Japan), followed by a streptavidin–horseradish peroxidase conjugate (Nichirei). Bound antibody was visualized with the use of the substrate 3,3′-diaminobenzidine (Sigma-Aldrich). Sections were counterstained with Mayer’s hematoxylin. For immunofluorescent staining, slides were incubated with the primary antibody (anti-Ki67, 1:300; together with chicken anti-MCT1, 1:200) overnight at 4 °C, followed by incubation with the fluorescence-conjugated secondary antibody (1:200) for 1 h. After washing, the sections were mounted with ProLong Gold Antifade with DAPI (Thermo Fisher Scientific). Images were acquired using a confocal microscope (Carl Zeiss, LSM 700, Oberkochen, Germany).

Immunofluorescence staining of UCP1 in the MEFs was performed as previously reported^3^, with a slight modification. Briefly, WT and Tg MEFs were differentiated on chamber slides. The cells were then washed twice with PBS and fixed with 10% formalin in PBS for 20 min at room temperature. Cells were washed three times with PBS and exposed to 5% glycine in PBS. After washing three times in PBS, cells were permeabilized with 5% acetic acid in ethanol for 10 min at −20 °C. Cells were then washed three times with PBS and incubated with 8% BSA in PBS for 1 h at room temperature, followed by incubation with antisera against UCP1 (1:800) overnight at 4 °C. Cells were washed three times with PBS prior to incubation with Alexa Fluor 488-labeled secondary antibodies (1:200) for 1 h at room temperature. After washing, cells were stained with DAPI and mounted with ProLong Gold Antifade. Images were acquired using a fluorescence microscope (Keyence, BZ-X700, Tokyo, Japan).

### Western blotting

Tissue specimens were homogenized in Tris-ethylenediaminetetraacetic acid (EDTA) buffer (10 mM Tris and 1 mM EDTA, pH 7.4). After centrifugation at 800 × g for 10 min at 4 °C, the resulting supernatant obtained as total protein was used for western blotting analysis. In brief, the protein was separated by SDS-PAGE and transferred to a polyvinylidine fluoride membrane (Immobilon; Millipore, Tokyo, Japan). After blocking with 5% skimmed milk (Morinaga Milk Industry Co., Tokyo, Japan), the membrane was incubated with a primary antibody overnight. The bound antibody was visualized using an enhanced chemiluminescence system (GE Healthcare UK Ltd., Little Chalfont, Bucks, UK) using a horseradish peroxidase-linked secondary antibody. UCP1 content in fat tissue was calculated using the data from western blotting and the total protein content.

### RNA analysis

Total RNA was extracted using RNAiso (Takara Bio, Shiga, Japan) according to the manufacturer’s protocol. The mRNA levels were measured quantitatively by real-time RT-PCR using the respective cDNA fragment as a standard, and normalized using β-actin mRNA as an internal standard. Briefly, 2 µg of total RNA was reverse transcribed using an oligo (dT) 15-adaptor primer and M-MLV reverse transcriptase (Thermo Fisher Scientific). Real-time PCR was performed using a fluorescence thermal cycler (LightCycler system; Roche Diagnostics, Mannheim, Germany), with SYBR Green (FastStart Essential DNA Green Master, Roche Diagnostics) employed as a double-strand DNA-specific dye, in accordance with the manufacturer’s protocol. Primers used are listed in Supplementary Table 1.

### DNA content

DNA content was assessed using a fluorometric method with bisbenzimidazole (Hoechst no.33258), as described previously^45^.

### Data analysis

Values are expressed as the mean ± standard error of the mean (SE). Statistical analyses were performed using the two-tailed unpaired Student’s t-test or one-way factorial analysis of variance (ANOVA) followed by the Tukey–Kramer *post-hoc* test.

## Electronic supplementary material

Supplementary Information
