# Supplementary material for: Cell-cycle arrest in mature adipocytes impairs BAT development but not WAT browning, and reduces adaptive thermogenesis in mice
Source: Sci Rep. 2017 Jul 27;7:6648. doi: 10.1038/s41598-017-07206-8 (PMC5532220; doi:10.1038/s41598-017-07206-8)
Supplement: Supplementary file 1 — Supplementary Information [file 41598_2017_7206_MOESM1_ESM.pdf]

Supplementary Information

Cell-cycle arrest in mature adipocytes impairs BAT development but not WAT browning, and reduces adaptive thermogenesis in mice

Yuko Okamatsu-Ogura[1,6*], Keigo Fukano[1,6], Ayumi Tsubota[1], Junko Nio-Kobayashi[2], Kyoko Nakamura[3], Masami Morimatsu[4], Hiroshi Sakaue[5], Masayuki Saito[1], Kazuhiro Kimura[1]

[1]Department of Biomedical Sciences, Graduate School of Veterinary Medicine, Hokkaido University, Sapporo 060-0818, Japan
[2]Laboratory of Histology and Cytology, Graduate School of Medicine, Hokkaido University, Sapporo 060-8638, Japan
[3]Pharmaceutical Research and Technology Institute, Kindai University, Osaka 577-8502, Japan
[4]Department of Disease Control, Graduate School of Veterinary Medicine, Hokkaido University, Sapporo 060-0818, Japan
[5]Department of Nutrition and Metabolism, Institute of Health Biosciences, Tokushima University Graduate School, Tokushima 770-8503, Japan.
[6]These authors equally contributed to this study.

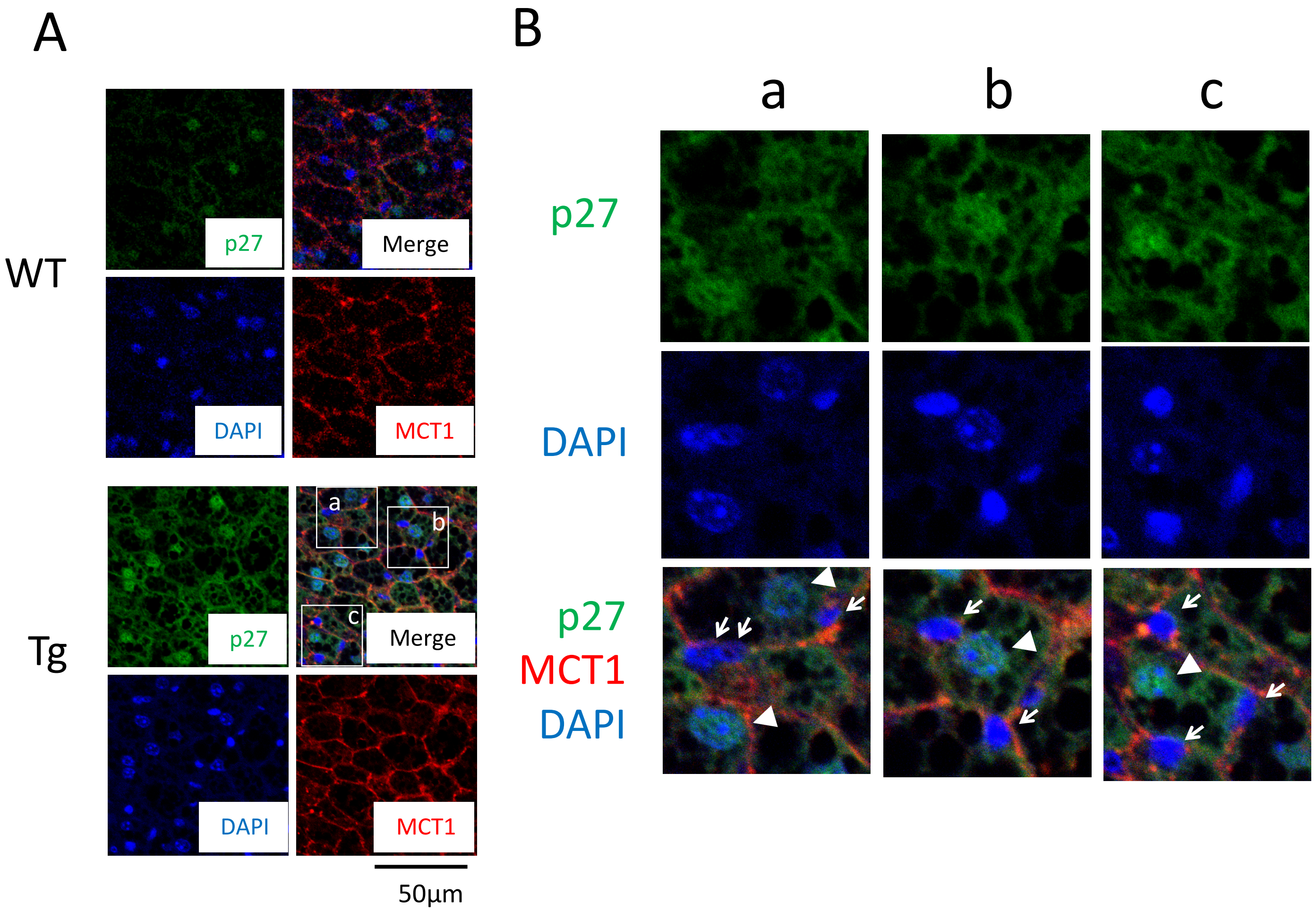

Supplementary figure S1. Localization of p27 in BAT of WT and aP2-p27 Tg mice

(A) Immunofluorescent staining of MCT1 (red) and p27 (green) was conducted in BAT of WT and aP2-p27 Tg mice. Nuclei were stained with DAPI (blue), MCT1 was detected on cell membrane of brown adipocytes. In both mice, p27 protein was detected in cytoplasm and nucleus of brown adipocyte, with higher intensity in nucleus, and the expression level was higher in Tg BAT than in WT BAT.

(B) Enlarged images of BAT of Tg mice. Two types of nuclei were found: one is large and round-shaped and dissociated from MCT1-positive membrane (arrowheads), and cells with such type of nucleus were identified as brown adipocytes. The other is flat-shaped nuclei distributed between the MCT1-positive cell membranes (arrows), and cells with such nucleus were identified as stromal-vascular (SV) cells. p27 expression was observed in nuclei of most of the brown adipocytes, but not in that of SV cells.

[method]

Deparaffinized sections at 5-µm thickness were incubated with PBS containing 0.3% TritonX-100 for 30 min and then with 10% normal goat serum for 1 h. After washing with PBS for 10 min twice, slides were incubated with the primary antibodies (mouse anti-p27, 1:200 together with chicken anti-MCT-1, 1:200) overnight at 4 ℃, followed by incubation with fluorescence-conjugated second antibody (1:200) for 1 h. After washing, the sections were mounted with ProLong Gold Antifade Mountant with DAPI (Thermo Fisher Scientific). Images were acquired using a confocal microscope (Carl Zeiss LSM 700).

Supplementary Table1 Primers used in Real-time PCR

UCP1	Forward	5’ - GTG AAG GTC AGA ATG CAA GC - 3’
	Reverse	5’ - AGG GCC CCC TTC ATG AGG TC - 3’
COX IV	Forward	5’ – TGA GCC TGA TTG GCA AGA GA - 3’
	Reverse	5’ – CGA AGC TCT CGT TAA ACT GG - 3’
CIDE-A	Forward	5’ – CTT ATC AGC AAG ACT CTG GAT G - 3’
	Reverse	5’ – GAA GGT GAC TCT GGC TAT TC - 3’
PGC-1a	Forward	5’ – GTG TGG AAC TCT CTG GAA CT - 3’
	Reverse	5’ – GCG TAC AAC TCA GAT TGC TC - 3’
Dio2	Forward	5’ – CAG TGT GGT GCA CGT CTC CAA TC - 3’
	Reverse	5’ – TGA ACC AAA GTT GAC CAC CAG - 3’
ß-actin	Forward	5’ - TCG TTAC CAC AGG CAT TGT GAT - 3’
	Reverse	5’ - TGC TCG AAG TCT AGA GCA AC - 3’
